# Production of hydroxycinnamoyl-shikimates and chlorogenic acid in *Escherichia coli:* production of hydroxycinnamic acid conjugates

**DOI:** 10.1186/1475-2859-12-15

**Published:** 2013-02-05

**Authors:** Bong-Gyu Kim, Woo Dam Jung, Hyejung Mok, Joong-Hoon Ahn

**Affiliations:** 1 Department of Bioscience and Biotechnology, Bio/Molecular Informatics Center, Konkuk University, Seoul, 143-701, Republic of Korea

**Keywords:** Chlorogenic acid, Hydroxycinnamic acid, Hydroxycinnamate-CoA quinate transferase, Hydroxycinnamate-CoA shikimate transferase

## Abstract

**Background:**

Hydroxycinnamates (HCs) are mainly produced in plants. Caffeic acid (CA), *p*-coumaric acid (PA), ferulic acid (FA) and sinapic acid (SA) are members of the HC family. The consumption of HC by human might prevent cardiovascular disease and some types of cancer. The solubility of HCs is increased through thioester conjugation to various compounds such as quinic acid, shikimic acid, malic acid, anthranilic acid, and glycerol. Although hydroxycinnamate conjugates can be obtained from diverse plant sources such as coffee, tomato, potato, apple, and sweet potato, some parts of the world have limited availability to these compounds. Thus, there is growing interest in producing HC conjugates as nutraceutical supplements.

**Results:**

Hydroxycinnamoyl transferases (HCTs) including hydroxycinnamate-CoA shikimate transferase (HST) and hydroxycinnamate-CoA quinate transferase (HQT) were co-expressed with 4-coumarateCoA:ligase (4CL) in *Escherichia coli* cultured in media supplemented with HCs. Two hydroxycinnamoyl conjugates, *p*-coumaroyl shikimates and chlorogenic acid, were thereby synthesized. Total 29.1 mg/L of four different *p*-coumaroyl shikimates (3-*p*-coumaroyl shikimate, 4-*p*-coumaroyl shikimate, 3,4-di-*p*-coumaroyl shikimate, 3,5-di-*p*-coumaroyl shikimate, and 4,5-di-*p*-coumaroyl shikimate) was obtained and 16 mg/L of chlorogenic acid was synthesized in the wild type *E. coli* strain. To increase the concentration of endogenous acceptor substrates such as shikimate and quinate, the shikimate pathway in *E. coli* was engineered. A *E. coli aroL* and *aroK* gene were mutated and the resulting mutants were used for the production of *p*-coumaroyl shikimate. An *E. coli aroD* mutant was used for the production of chlorogenic acid. We also optimized the vector and cell concentration optimization.

**Conclusions:**

To produce *p*-coumaroyl-shikimates and chlorogenic acid in *E. coli*, several *E. coli* mutants (an aroD mutant for chlorogenic acid production; an aroL, aroK, and aroKL mutant for *p*-coumaroyl-shikimates production) were made and each mutant was tested using an optimized construct. Using this strategy, we produced 235 mg/L of *p*-coumaroyl-shikimates and 450 mg/L of chlorogenic acid.

## Background

Hydroxycinnamates (HCs) such as caffeic acid (CA), *p*-coumaric acid (PA), ferulic acid (FA) and sinapic acid (SA) are mainly produced in plants. Because HCs are anti-oxidants that can scavenge free radicals, it has been suggested that consumption of HCs might prevent cardiovascular disease and some types of cancer. In addition, several biological functions of HCs have been described, such as protection against side effects of chemotheraphy and anti-osteoclast activity [1].

In plants, HCs are usually conjugated with other compounds such as quinic acid, shikimic acid, malic acid, anthranilic acid, and glycerol [2]. Chlorogenic acid, the conjugate of CA and quinate, is abundant in coffee, fruits, and vegetables, and is the primary source of CA in the human diet [3]. For regular consumers of coffee in Western countries, the major dietary phenolics are chlorogenic acids [4].

The solubility of HCs is increased through thioester conjugation to various compounds. Although there is growing evidence for their beneficial health effects, the fruits and vegetables that contain high levels of HC conjugates are of limited availability in some parts of the world. Thus, there is growing interest in producing HC conjugates as nutraceutical supplements. The primary strategy for obtaining diverse phytochemicals is by extracting them from plants. However, this approach faces several obstacles such as a limited availability of plant materials and high costs of extraction and purification. Alternatively, they can be produced in microorganisms. Phytochemicals such as flavonoids and stilbenes have been produced using microorganisms such as *Escherichia coli* and *Saccharomyces cerevisiae*[5-7].

Biosynthesis of HC conjugates is mediated by hydroxycinnamoyl transferases (HCTs), which use the coenzyme A thioester of HCs as a donor and various compounds such as quinate, and shikimate as HC acceptors. The formation of coenzyme A thioester with HC is catalyzed by 4-coumaric acid:CoA ligase (4-CL) [8]. Thus, genes encoding 4-CL and HCT, are essential for producing HC conjugates from HC. HCTs from several plants have been characterized [8-12]. HCTs show specificity for not only the acyl group donor but also the acyl group acceptor. When the acyl group acceptors are shikimate and quinate, the resulting conjugates are *p*-coumaroyl-*O*-shikimate and chlorogenic acid, respectively [13,14].

*E. coli* is a good system for producing plant secondary metabolites including phytochemicals produced through the phenylpropanoid pathway [15]. We attempted to produce HC conjugates using *E. coli*. Shikimate and 3-dehydroquinate, which are acyl group acceptors for hydroxycinnamate-CoA shikimate transferases (HST) and hydroxycinnamate-CoA quinate transferases (HQT), respectively, can be synthesized through the shikimate pathway of *E. coli*[16,17]. Thus, it should be possible to alter the shikimate pathway to increase the concentrations of shikimate and quinate. However, the acyl donor (PA or CA) is a unique product of plants [18]. In order to produce HCs in *E. coli*, coexpression of several genes in *E. coli* is necessary, which leads to metabolic load. Therefore addition of exogenous HCs may circumvent this problem. In this report, we introduced either HST and 4CL (for the production of CA-shikimate)*,* or HQT and 4CL (for the production of chlorogenic acid) into *E. coli* mutants that accumulate either shikimate or 3-hydroquinate. When the engineered cells were fed exogenous HCs, a high yield of HC-shikimate and HC-quinate were obtained.

## Results

### Construction and selection of the optimum expression vector for the synthesis of HC-shikimate

**Figure 1:**
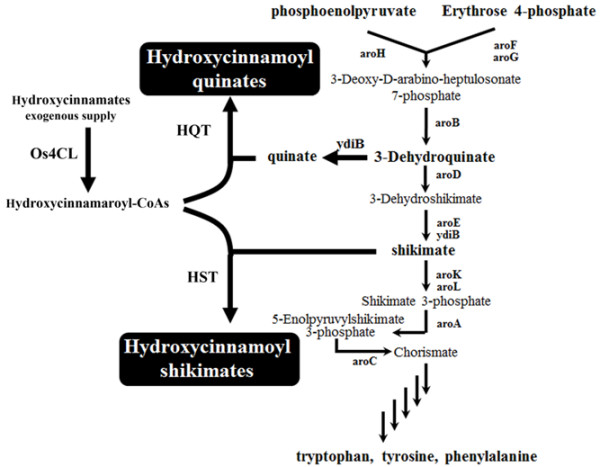
**Schematic diagram showing the shikimate pathway of *Escherichia coli *and production of hydroxycinnamoyl quinate and hydroxycinnamoyl shikimate in *E. coli.***

HC-shikimate was synthesized from HC-CoA and shikimate using HST (Figure 1). Two genes, HST from *Nicotiana tabacum* (*NtHST*) and 4CL from *Oryza sativa* (*Os4CL*), were subcloned into an *E. coli* expression vector. Os4CL converts the HCs into the corresponding HC-CoAs. Os4CL has a better catalytic efficiency than other 4CLs [19]. NtHST uses HC-CoAs such as *p*-coumaroyl-CoA, caffeoyl-CoA, and feruloyl-CoA as acyl donors and shikimate as an acyl acceptor to produce HC-shikimate. NtHST was the first characterized HST and only a few number of HST has been biochemically characterized. NtHST also uses quinate as an acyl acceptor [9]. Both genes (*NtHST* and *Os4CL*) were subcloned into three different *E. coli* expression vectors, each of which has a different copy number in *E. coli*. Each construct (pA-NtHST-Os4CL, pC-NtHST-Os4CL, or pE-NtHST-Os4CL; see Table 1) was transformed into *E. coli* BL21(DE3) cells, and tested for its ability to produce HC-shikimate when incubated with exogenous PA. As a control, the empty vector was transformed into *E. coli*. HCs including PA can enter into *E. coli* via the 4-hydroxyphenylacetate permease (HpaX transporter) [20] and small phenolic compounds can be exported from *E. coli* into the culture medium. The transformant harboring pC-NtHST-Os4CL produced the highest concentration of HC-shikimate derivatives (29.1 mg/L), whereas the titers of HC-shikimate derivatives produced by the transformants harboring pA-NtHST-Os4CL or pE-NtHST-Os4CL were 24.4 and 27.4 mg/L, respectively. However, only PA was observed in the transformant harboring the empty vector. According to ANOVA test, a significant difference in PA-shikimates production at P=0.01 was shown between pC-NtHST-Os4CL and pA-NtHST-Os4CL or between pE-NtHST-Os4CL and pA-NtHST-Os4CL, but not between pC-NtHST-Os4CL and pE-NtHST-Os4CL. The construct present in the pCDF vector (pC-NtHST-Os4CL) was used in further studies because this construct has a lower copy number than pE-NtHST-Os4CL. Therefore, pC-NtHST-Os4CL is expected to impose a lower metabolic load on *E. coli*.

**Table 1:** **Plasmids, *Escherichia coli *strains, and primers used in this study**

**Plasmids or *E. coli *strains or Primers**	**Relevant properties or genetic marker**	**Source or reference**
Plasmids		
pCYCDuet	P15A ori, Cm^r^	Novagen
pCDFDuet	CloDE13 ori, Str^r^	Novagen
pETDuet	f1 ori, Amp^r^	Novagen
pA-NtHST-Os4CL	pACYCDDuet carrying *NtHST* from *N. tobacco* and *4CL* from *O. sativa*	This study
pC-NtHST-Os4CL	pCDFDuet carrying *NtHST* from *N. tobacco* and *4CL* from *O. sativa*	This study
pE-NtHST-Os4CL	pETDuet carrying NtHST from *N. tobacco* and *4CL* from *O. sativa*	This study
pC-NtHQT-Os4CL	pCDFDuet carrying *NtHQT* from *N. tobacco* and *4CL* from *O. sativa*	This study
pA-EcydiB	pACYC carrying *ydiB* from *E. coli*	This study
Strains		
BL21 (DE3)	F^-^*ompT hsdS*_*B*_(r_B_- m_B_-) *gal dcm lon* (DE3)	Novagen
B-100	BaroD carrying pCDF-Duet	This study
B-101	BaroD carrying pC-NtHQT-Os4CL	This study
B-102	BaroD carrying pC-NtHQT-Os4CL and pA-EcydiB	This study
BaroD	BL21(DE3) *ΔaroD*::*FRT-kan*^*R*^*-FRT*	This study
BaroK	BL21(DE3) *ΔaroK*::*FRT-kan*^*R*^*-FRT*	This study
BaroL	BL21(DE3) *ΔaroL*::*FRT-kan*^*R*^*-FRT*	This study
BaroKL	BL21(DE3) *ΔaroK*::*FRT ΔaroL*::*FRT-kan*^*R*^*-FRT*	This study
Primers		
NtHST-F^1^	AACATATGAAGATCGAAGTGAAAGAAT (NdeI site is underlined)	
NtHST-R^2^	AACTCGAGTCAAAAGTCATACAAGAACTTC (XhoI site is underlined)	
NtHQT-F	AAGATATCCATGGGAAGTGAAAAAATGATGA (EcoRV site is underlined)	
NtHQT-R	AAGGTACCTCAAAATTCATACAAATACTT (KpnI site is underlined)	
ydiB-F	ATGAATTCGATGGATGTTACCGCAAAATAC (*EcoR*I site is underlined)	
ydiB-R	CATGCGGCCGCTCAGGCACCGAACCCCATG (*Not*I site is underlined)	
aroK-F	gctgtcttttttacgctaatcttacccggtgatttatcgccagagcggtgaattaaccctcactaaagggcg	
aroK-R	cccgcagacgagtgtatataaagccagaattagttgctttccagcatgtgtaatacgactcactatagggctc	
aroL-F	atgacaccggctttcgccgcattgcgacctattggggaaaacccacgatgaattaaccctcactaaagggcg	
aroL-R	gatgaacgttaagtataggcgctcgaaaatcaacaattgatcgtctgtgctaatacgactcactatagggctc	
aroD-F	tggggttcggtgcctgacaggctgaccgcgtgcagaaagggtaaaaaatgaattaaccctcactaaagggcg	
aroD-R	gggaggatattcccgccgaaatattattgcttatgcctgatgtaaaatagttaatacgactcactatagggctc	
aroK-check-F	cgctgcctgcgttccatgat	
aroL-check-F	cgcggagctggagaagtggt	
aroK or aroL check-R	taatacgactcactatagggctc	
aroD-check-F	ggcaaggggctgaacagttc	
aroD-check-R	gggaggatattcccgccgaa	

^1^F means forward primer.

^2^R means reverse primer.

**Figure 2:**
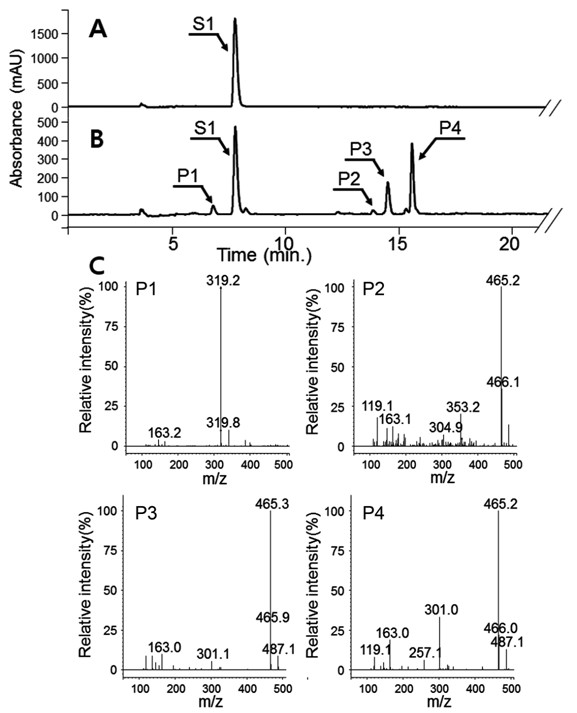
**High performance liquid chromatography analysis of biotransformation of *p*-coumaric acid in BL21 (DE3) harboring *Os4CL *and *NtHST. *A**, *p*-coumaric acid (S1); **B**, reaction products of *p*-coumaric acid (P1 - P4); **C**, mass spectrometry (MS) data for the four reaction products. The mass spectrometer was operated in negative mode. The molecular masses of *p*-coumaric acid and shikimic acid are 164 Da and 174 Da, respectively.

Although each vector exhibited varying biotransformation efficiencies, they all had the same reaction profiles. As shown in Figure 2, four new peaks appeared (P1 - P4). The molecular mass of P1 was 320.2 Da, indicating that one molecule of PA was attached to shikimate probably at the 5'-hydroxyl group of shikimate according to enzymatic studies [9,10]. In contrast, the molecular mass of the other three reaction products (P2 - P4) was 466.2 Da. Because the molecular mass of PA is 164 Da and that of shikimate is 174 Da, the three reaction products must be conjugates of two PA molecules and one shikimate molecule. *p*-Coumaroyl shikimates are not commercially available and the structures of the reaction products was determined using nuclear magnetic resonance spectroscopy (NMR). By the help of the HMBC and HMQC experiments (Additional file 1: Figure S1, S2), 14 ^13^C peaks were determined, so that P1 was considered to be a conjugate of PA and shikimate. Even this structure contains 16 carbons, two set symmetric carbons of PA provide only two carbon peaks. Based on the HMBC spectrum (Additional file 1: Figure S1), the carboxyl group of PA was connected to the hydroxyl group of shikimate. Three isomers, the carboxyl group of PA connected to 3"-OH or 4"-OH or 5"-OH of shikimate, were possible. In the current NMR experiments, we could not determine the connected position. The name of the product P1 is (E)-3,4-dihydroxy-**5**-(3-(4-hydroxyphenyl)acryloyloxy)cyclohex-1-enecarboxylic acid where the bold number **5** could be switched with 3 or 4.

Based on the molecular masses of P2, P3, and P4 (Figure 2C), they contained two PAs and one shikimate. Two PAs can be connected to 3"-OH or 4"-OH or 5"-OH of SA, so that there can be three isomers, i.e. 3"-OH/4"-OH, 3"-OH/5"-OH, or 4"-OH/5"-OH. We could not determine which produce was which isomer. By comparison to a previous report [21], P3 and P4 are likely to be 3,5-di-*p*-coumaroyl shikimate and 4,5-di-*p*-coumaroyl shikimate, respectively. The remaining peak, P2 is therefore likely to be 3,4-di-*p*-coumaroyl shikimate.

Using *E. coli* harboring pC-NtHST-Os4CL, we determined the best acyl donor among CA, PA, and FA. In a previous study, recombinant NtHST protein most efficiently used caffeoyl-CoA as an acyl donor [9]. In this study, 1 mM of each HC was added to the same number of cells, and biotransformation was performed for 6 h. PA was the most effective acyl donor, producing 23.9 mg/L of *p*-coumaroyl shikimate. The amounts of feruloyl shikimate and caffeoyl shikimate produced were 3.8 mg/L, and 3.1 mg/L, respectively. Taken together, these results indicate that the highest amount of HC-shikimate was obtained with pC-NtHST-Os4CL as the construct and PA as the substrate.

### Engineering *E. coli* to increase production of PA-shikimate conjugates

**Figure 3:**
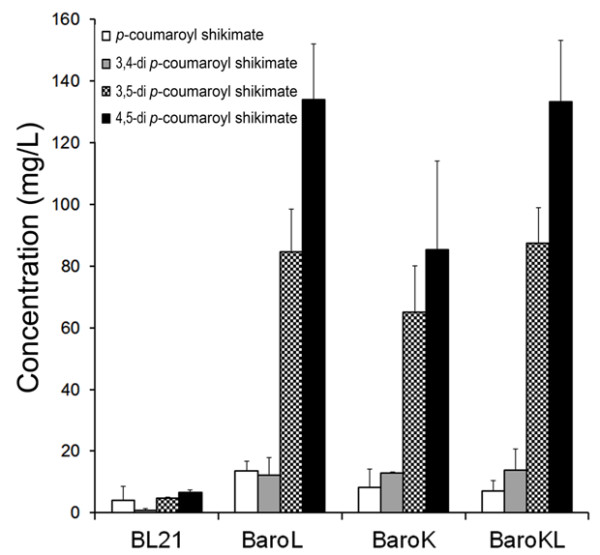
**Production of hydroxycinnamic shikimates in different *E. coli *strains.** Product 1 contains one molecule of hydroxycinnamate attached to shikimate, while products 2–4 contain two molecules of hydroxycinnamate attached to shikimate. Error bars indicate mean values ± SD from three independent experiments. Product 1 is 3 or 4-*p*-coumaroyl shikimate; The structures of product 2, product 3, and product 4 are likely to be 3,4-di-*p*-coumaroyl 3,5-di-*p*-coumaroyl shikimate, and 4,5-di-*p*-coumaroyl shikimate, respectively.

Because PA was added to the *E. coli* culture as an acyl donor, it was not a limiting factor for the production of PA-shikimate conjugates. However, *E. coli* uses endogenous shikimate as an acyl group acceptor. Thus, limiting shikimate availability would cause a bottleneck in the production of PA-shikimate conjugate. Previous studies showed that deletion of *aroK* and/or *aroL* in *E. coli* results in increased shikimate accumulation [22-24]. We created an *E. coli aroL* deletion mutant (BaroL), an *aroK* deletion mutant (BaroK) and an *aroK/aroL* double mutant (BaroKL) by gene replacement. pC-NtHST-Os4CL was transformed into each mutant and into wild type. BaroL showed the highest productivity (approximately 235 mg/L), which is approximately 15-fold higher than BL21 (16 mg/L). BaroKL and BaroK produced 228 mg/L and 171 mg/L of PA-shikimates, respectively (Figure 3). According to ANOVA test, PA-shikimates production was significantly different at P=0.01 level among BL21, BaroK, and BaroL. However, there was no difference between BaroL and BaroKL at this level of significance.

Next, production of PA-shikimate was optimized using BaroL harboring pC-NtHST-Os4CL. The optimum cell concentration was determined by varying the cell concentration from OD_600_ 1 to 5 in the presence of 1 mM PA. Production of PA-shikimate peaked at an OD_600_ of 3, at which cell concentration the yield of PA-shikimate was approximately 235 mg/L. At OD_600_ = 1, or 2, the yield was approximately 94 mg/L, and 169 mg/L, respectively. Above OD_600_ = 3, the production of PA-shikimate decreased and was approximately 188 or 103.7 mg/L at OD_600_ = 4 or 5, respectively.

**Figure 4:**
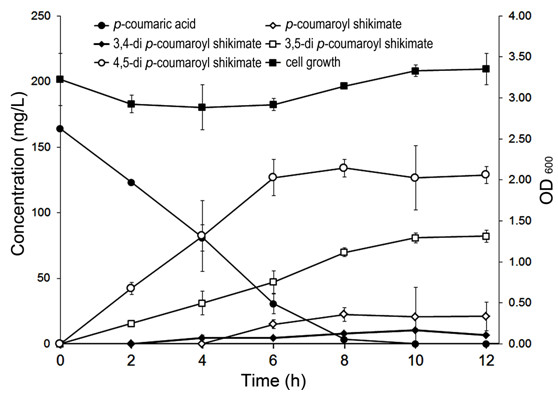
**Production of hydroxycinnamic shikimates by biotransformation using BaroL harboring pC-NtHST-Os4CL.** Error bars indicate mean values ± SD from three independent experiments.

Using BaroL harboring pC-NtHST-Os4CL at a cell concentration of OD_600_ 3, we monitored the production of HC-shikimate over 12 h. The maximum amount of di-*p*-coumaroyl shikimates was produced at 8 h (134 mg/L 3,5-di-*p*-coumaroyl shikimate, 70 mg/L 4,5-di-*p*-coumaroyl shikimate, 8 mg/L of 3,4-di-*p*-coumaroyl shikimate, and 23 mg/L of 5-*p*-coumaroyl shikimate). Therefore, a total of 235 mg/L of PA-shikimates was produced. At 8 h, the amount of 4,5-di-*p*-coumaroyl shikimate and 3,5-di-*p*-coumaroyl shikimate was reduced, but the amount of 5-*p*-coumaroyl shikimate reached a maximum (Figure 4). It seems that 5-*p*-coumaroyl shikimate was immediately converted to either 3,5-di-*p*-coumaroyl shikimate or 4,5-di-*p*-coumaroyl shikimate before 8 h.

### Production of HC-quinate in *E. coli*

**Figure 5:**
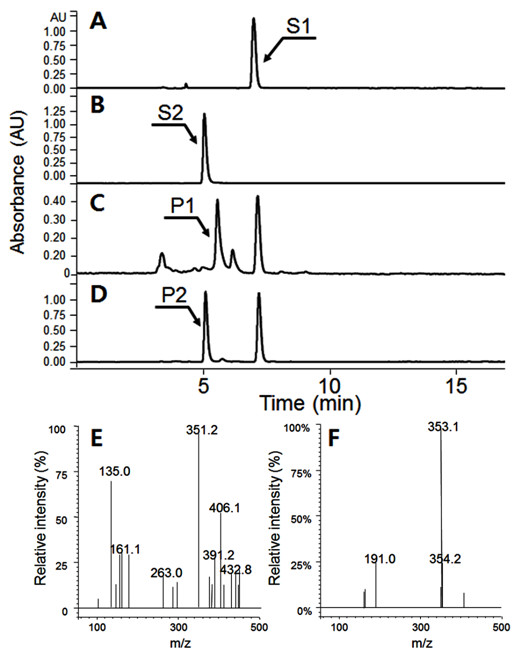
**Production of chlorogenic acid in *E. coli*. A**, caffeic acid (S1); **B**, chlorogenic acid (S2); **C**, reaction product of caffeic acid obtained from strain B-101; **D**, reaction product of caffeic acid obtained from strain B-102; **E**, MS/MS profile of P1; F, MS/MS profile of P2.

We used an acyltransferase that is specific for quinate to produce HC-quinate. Hydroxycinnamoyl-CoA quinate transferase from *N. tobacco* (NtHQT) was shown to be more specific for quinate than for shikimate [9,25]. Os4CL mediates formation of coenzyme A thioester with HC. To produce HC-quinate in *E. coli*, two genes, *NtHQT* and *Os4CL,* were subcloned into an *E. coli* expression vector. The resulting construct, pC-NTHQT-Os4CL was transformed into BL21(DE3), and the transformant was used for biotransformation of CA. However, no reaction product was detectable. The amount of quinate or dehydroquinate in *E. coli* may not be high enough to drive the production of chlorogenic acid. Therefore, we created strain BaroD, which should accumulate higher levels of dehydroquinate and/or quinate, in order to overcome this potential limitation. The strain BaroD was used as a host for pC-NTHQT-Os4CL and the resulting transformant was called B-101 (Table 1). As control, empty pCDFDuet was transformed into the strain BaroD and this transformant was called B-100 (Table 1). Each transformant was used for biotransformation of CA. The culture medium of B100 became a brown color after 8 h. Analysis of the reaction mixture using HPLC after 8 h incubation showed a trace level of caffeic acid. In addition, no detectable new product was observed. The decrease of caffeic acid during biotransformation is associated with the formation of *O*-quinone and its polyaromatic derivatives [26]. However, analysis of the biotransformation mixture from strain B-101 showed a new peak (Figure 5C) and the color of the culture medium did not change. Once caffeic acid is converted into another compound, it cannot polymerize and therefore the color of the culture medium does not change. The molecular mass of the product was 352 Da, which was 2 Da less than the predicted molecular mass of the thioester of quinate and CA (Figure 5C and E). Based on the molecular weight of the reaction product and the known intermediates of the *E. coli* shikimate pathway, it is like that 3-dehydroquinate and not quinate was attached to CA. NtHQT utilized 3-dehydroquinate as an acceptor and caffeoyl-CoA as a donor to make caffeoyl-3-dehydroquinate. Expression of *ydiB* from *E. coli*, which converts dehydroquinate into quinate, also increased the production of HC ester yield because NtHQT was likely to have higher affinity for quinate than for dehydroquinate as acyl group acceptor.

The protein product of *ydiB* converts 3-dehydroquinate into quinate [27]. *ydiB* was overexpressed in *E. coli* strain B-101 to make strain B-102. Biotransformation of CA by strain B-102 resulted in a new product with an identical HPLC retention time and molecular mass (354 Da) as chlorogenic acid (Figure 5D, F). The MS/MS spectrum of the reaction product matched authentic chlorogenic acid. This indicates that ydiB converts 3-dehydroquinate into quinate, which was then utilized for the production of chlorogenic acid.

PA and FA were also tested as acyl-group donors. CA was the best acyl-group donor followed by PA and FA. After 24 h, the amounts of caffeoyl-quinate, *p*-coumaroyl-quinate, and feruloyl-quinate were 450 mg/L, 323.7 mg/L, and 216 mg/L, respectively.

**Figure 6:**
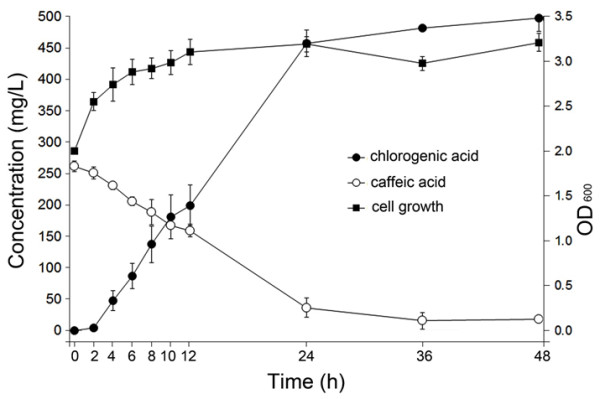
**Production of chlorogenic acid using the *E. coli *strain B-106.**

The production of chlorogenic acid was monitored further in strain B-102. After induction, 1.1, 1.3, 1.5, 1.7, or 2.0 mM CA was added to the culture. After 24 h, almost all of the CA was converted into chlorogenic acid in the reaction mixtures supplied with 1.1, 1.3, or 1.5 mM of CA. However, at a concentration of 1.7 or 2.0 mM, some CA remained in the mixture, and even after a longer incubation time, the remaining caffeic acid was not converted into chlorogenic acid. However, the color of the mixture became dark brown due to the oxidation/polymerization of CA. Therefore, we monitored chlorogenic acid production at a CA concentration of 1.5 mM. As shown in Figure 6, production of chlorogenic acid continued to increase until 24 h, while the CA content decreased. Approximately 450 mg/L of chlorogenic acid was produced at 24 h.

## Discussion

Anthocyanins, flavonoids, stilbene, and other compounds of plant origins have been biologically synthesized using engineered *E. coli* strains [7,15]. Most of these efforts involved introduction of new genes with high activity into *E. coli*. In addition, cofactor supplementation and reducing equivalents have been enhanced to produce natural compounds using engineered *E. coli*[6,28]. The goal of this study was to produce PA-shikimate and chlorogenic acid in *E. coli*. We also engineered the shikimate pathway of *E. coli* to accumulate the acyl group acceptors, shikimate and quinate, which led to the increased production of PA-shikimates and chlorogenic acid.

During biotransformation, two PA molecules are attached to shikimate, whereas only one molecule of CA is attached to quinate. In plants, one molecule of CA is bound to one molecule of shikimate. However, diverse HC-quinate conjugates, including those in which two or three identical HCs are bound to quinate, and two or three different HCs are bound to quinate, were found in coffee (*Coffea robusta*) and sweet potato (*Ipomonea batatas*) [21,29]. In these plants, the mono-esters are present as major components whereas di- and tri-esters are present as minor components. NtHST and NtHQT may have different enzymatic properties from HCTs from coffee or sweet potato because di-*p*-coumaroyl skimates and cholorgenic acid were synthesized using NtHST and NtHQT. One possible scenario is that the substrate binding pocket of NtHQT may be narrower than that of NtHST. Thus, HC-quinate may not fit into the substrate binding pocket of NtHQT for the second round of acyl transfer reaction. Longer incubation of the recombinant NtHST enzyme with PA-CoA and shikimate resulted in the production of di-PA-shikimate (data not shown). The production of di-PA-shikimate was also observed with HST from *Populus euramericana*[13]. Using the properties of NtHST, diverse HC-shikimate conjugates could possibly be obtained and feeding of different concentrations of various HCs into the *E. coli* culture medium may result in production of various forms of HC-shikimate conjugates. Any biological activity including anti-oxidant activity of di-PA-shikimate compared to PA-shikimate has not been tested. However, 8-*O*-4-diferulic acid showed better antioxidant activity than ferulic acid [30]. 

*p*-Coumaric acid can be synthesized from tyrosine by the action of tyrosine ammonia lyase (TAL). Therefore, synthesis of *p*-coumaroyl-shikimate or chlorogenic acid from glucose might be possible if a gene econding TAL were expressed into *E. coli* harboring *NtHST* and *Os4CL* or into *E. coli* harboring *NtHQT*, *Os4CL*, *ydiB*, and *Sam5* (Sam5 converts *p*-coumaric acid into caffeic acid [31]), respectively. However, it is expected that only a tiny amount of reaction product would be produced from glucose in a wild type strain because quinate and shikimate are not accumulated in the wild type *E. coli*. Use of strain BaroD or BaroL is not also feasible because tyrosine is not synthesized in these strains. Thus, supplementation with either caffeic acid or tyrosine is needed to synthesize *p*-coumaroyl-shikimate or chlorogenic acid in *E. coli*.

Wild type *E. coli* expressing NtHQT and Os4CL did not produce detectable amounts of chlorogenic acid or caffeoyl-dehydroquinate after CA supplementation. In the wild type, dehydroquinate may have been rapidly converted into another downstream compound of the shikimate pathway instead of accumulating. Moreover, the expression level of *ydiB*, which converts dehydroquinate into quinate, was low. Only a small amount of quinate or dehydroquinate will be expected to be present in the cell in the absence of overexpression of *ydiB*[32]. ydiB and aroE mediate the conversion reaction not only from dehydroquinate to quinate but also from dehydroshikimate to shikimate. However, aroE prefers dehydroshikimate to dehydroquinate [22], whereas ydiB has nearly the same catalytic efficiency for dehydroshikimate and dehydroquinate [27,33]. Recent studies have shown that overexpression of *ydiB* does not increase shikimate, while overexpression of *aroE* increases conversion of dehydroshikimate to shikimate [32]. Therefore, we overexpressed *ydiB* to produce chlorogenic acid. In addition, strains BaroK, BaroL, and BaroKL expressing pC-NtHQT-Os4CL that were supplemented with CA produced chlorogenic acid only when ydiB was overexpressed (data not shown). This indicated that these mutants accumulate shikimate pathway intermediates such as dehydroquinate, dehydroshikimate and shikimate. These intermediates are converted into quiniate by ydiB.

## Conclusions

To produce the HC-conjugates in *E. coli*, several *E. coli* mutants including aroD, aroK, aroL, and aroK/L mutants were made and each mutant was tested using an optimized construct. For the production of HC-shikimates, an *E. coli aroL* mutant (B-aroL) was best and we produced 235 mg/L of HC-shikimates using B-aroL expressing *NtHST* and *Os4CL*, which is approximately 15-fold higher than wild type *E. coli* BL21 (16 mg/L). The wild type *E. coli* expressing *NtHQT* and *Os4CL* did not produce any detectable chlorogenic acid. By using *E. coli aroD* mutant (strain B-101), which accumulated 3-dehydroquinate, caffeoyl-3-dehydroquinate instead of chlorogenic acid, was synthesized. However, by expressing *ydiB* gene in strain B-101, which converted 3-dehyroquinate to quinate, chlorogenic acid production was dramatically increased up to 450 mg/L.

## Materials and methods

### Strains and reagents

The *E. coli* strains used in this study are listed in Table 1. *E. coli* BL21 (DE3) cells were used for recombinant protein production. *E. coli* DH5α cells were used for plasmid cloning. All restriction enzymes and T4 DNA ligase were purchased from Takara (Shiga, Japan). Polymerase chain reaction (PCR) amplification was performed using Hotstart Taq DNA polymerase (Qiagen, Hilden Germany). Reverse transcription was performed using Omniscript reverse transcriptase (Qiagen). *E. coli* was cultured in Luria-Bertani (LB) or M9 medium (plus 2% glucose) containing 50 μg/mL antibiotics, when necessary. *E. coli* expression vectors were purchased from Novagen (Madison, WI, USA).

### Construction of *E. coli* expression vector

The genes for hydroxycinnamate-CoA shikimate transferase (*NtHST*) [9] and hydroxycinnamate-CoA quinate transferase (*NtHQT*) from tobacco (*N. tabacum*) [26] were cloned using reverse-transcription polymerase chain reaction (RT-PCR). Total RNA was isolated from the leaves of one-month-old tobacco using Plant Total RNA Isolation Kit (Qiagen), and cDNA was synthesized using Omniscript reverse transcriptase (Qiagen) and oligo dT as a primer. PCR was carried out using primers designed on the basis of the published sequences (GenBank accession AJ507825 for *NtHST* and AJ582651 for NtHQT). The primers were listed in Table 1. The *Os4CL* gene, which was cloned and characterized previously from rice [19], was subcloned into the *Bam*HI/*Not*I sites of pACYCDuet, pCDFDuet, and pETDuet vectors (EMD Chemicals, Gibbstown, NJ, USA), and then the resulting *NtHST* PCR product was subcloned into a second cloning site, the *Nde*I/*Xho*I site of each vector. The NtHQT PCR product was subcloned into the *EcoR*V/*Kpn*I site of pCDFDuet, which contains Os4CL at *Nde*I/*Xho*I site. The resulting constructs are listed in Table 1.

Deletion of the *aroL* and *aroK* genes in *E. coli* BL21(DE3) was accomplished using the Quick and Easy Conditional Knockout Kit (Gene Bridges, Heidelberg, Germany). Briefly, the *aroL* gene or the *aroK* gene of *E. coli* BL21 (DE3) was replaced by the *ΔaroL* FRT-PGK-gb2-neo-FRT cassette or the *ΔaroK* FRT-PGK-gb2-neo-FRT cassette, respectively [34]. Deletion mutants were selected in Luria-Bertani (LB) medium containing 50 μg/mL kanamycin. Deletion of *aroL* or *aroK* was confirmed using PCR. The strains deleted in *aroL* or *aroK* were named BaroL and BaroK, respectively (Table 1). *ΔaroL* and *ΔaroK* double mutant (strain BaroKL in Table 1) was constructed using strain BaroL. The kanamycin cassette was removed in *ΔaroL* using an FLP expression plasmid, which removes the kanamycin selection marker from the chromosome. Removal of the kanamycin cassette was confirmed by PCR. Using *ΔaroL* as a host, the *aroK* gene was replaced by the *ΔaroK* FRT-PGK-gb2-neo-FRT cassette. The primer sequences for the *aroD, aroK,* and *aroL* deletion are shown in Table 1.

Shikimate/quinate dehydrogenase gene, *ydiB* was cloned using *E. coli* BL21 (DE3) genomic DNA as a template. Primers were designed based on the published sequence (NC_000913.2) and the sequences of primers were listed in Table 1. The PCR product was digested with *EcoR*I/*Not*I and subcloned into the corresponding site of pCDF-Duet1. The resulting constructs were confirmed by sequencing.

### Production of HC-shikimate conjugate in *E. coli*

Each construct was transformed into *E. coli* BL21 (DE3) strain or BaroL strain using electroporation with the BioRad MicroPulser Electroporation Apparatus (BioRad, Hercules, CA, USA). Overnight cultures of transformants were inoculated into LB medium containing 50 μg/mL of antibiotic at 37°C and cultured until the OD_600_ reached 0.8. Protein expression was induced by the addition of 1 mM isopropyl β-D-1-thiogalactopyranoside (IPTG), and growth was continued for another 20 h at 18°C. Cells were harvested and resuspended to obtain a cell concentration corresponding to OD_600_ of 3 in 2 mL of fresh M9 medium containing 2% glucose, 50 μg/ mL of antibiotics, 1 mM IPTG, and 1 mM PA in a test tube (14 mm × 145 mm) and then cultured at 30°C for 8 h. The supernatant was extracted twice using an equal volume of ethyl acetate, and the upper aqueous phase was dried using a Speed Vac at 30°C, dissolved in 60 μL of dimethylsulfoxide (DMSO), and analyzed using high-performance liquid chromatography (HPLC). Because *p*-coumaroyl shikimate is not commercially available, we used *p*-coumaric acid to generate a standard curve for quantitative analysis of the reaction products. The UV spectra of *p*-coumaric acid are distinguishable from those of *p*-coumaroyl shikimate but they are very similar and the absorption of the thioesters is usually shifted to longer wavelength by a few nm. This is subtle enough that it should be perfectly acceptable to use the free acids as quantitation standards for the thioesters.

To determine the maximum conversion rate using the optimized vector, protein expression was induced as described above. The cell concentration was adjusted to an OD_600_ of 3 in 25 mL of fresh M9 containing 2% glucose, 1 mM IPTG, and 50 μg/mL of antibiotics. The medium was supplemented with 1 mM PA. The reaction product (200 μL) was collected and extracted with ethyl acetate. The supernatant was dried and dissolved with 100 μl of dimethyl sulfoxide (DMSO). The sample was directly injected to HPLC for analysis. The mean and the standard error of the mean were calculated from triplicate experiments. Analysis of variance (ANOVA) was carried out using Tukey’s method with a significance level of P=0.01 using 2010 Microsoft Office Excel.

### Production of chlorogenic acid in *E. coli*

The construct for the production of chlorogenic acid was transformed into *E. coli* BL21(DE3) or BaroD cells. Induction of each construct was performed as described above. To determine the optimal gene construct, 200 μM PA was added to the growth medium.

To measure the production of chlorogenic acid in BaroD cells harboring pC-EcycdiB and pC-NtHQT-Os4CL (Table 1), the cell concentration was adjusted to an OD_600_ of 2.0, and CA was added to the medium to a final concentration of 1.5 mM. Production of chlorogenic acid was periodically monitored. The biotransformation was stopped by boiling for 5 min and the biotransformation product was centrifuged for 15 min at 13000 × g to remove the cell debris and other components prior to HPLC analysis. The quantification of the product was carried out using a standard curve generated with authentic chlorogenic acid (Sigma, MO, USA).

### Analysis of the metabolites

The metabolites were analyzed using a Varian HPLC equipped with a photo diode array (PDA) detector and a Varian C18 reversed-phase column (Varian, 4.60 × 250 mm, 3.5 μm particle size). The mobile phases consisted of 0.1% formic acid in water and acetonitrile. For chlorogenic acid, the program was: 20% acetonitrile at 0 min, 32% acetonitrile at 15 min, 90% acetonitrile at 17 min, 90% acetonitrile at 20 min, 20% acetonitrile at 21 min, and 20% acetonitrile at 26 min. To analyze hydroxycinnamoyl shikimate, the program was: 25% acetonitrile at 0 min, 40% acetonitrile at 10 min, 75% acetonitrile at 15 min, 90% acetonitrile at 22 min, 25% acetonitrile at 23 min, and 25% acetonitrile at 30 min. The flow rate was 1 mL/min, and the separation was monitored at 290 nm and 320 nm.

The molecular masses of the metabolites were determined using a Varian 500-MS ion trap spectrometer. Mass spectra were acquired simultaneously using an electrospray ionization source in negative ionization mode at 600 V. NMR spectrometry was done as described before [35].

## Competing interests

The authors declare that they have no competing interests.

## Authors’ contributions

JHA initiated and coordinated the project. BGK, JWD, HM, and JHA performed experiments, analyzed data and wrote the paper. All authors approved the final manuscript.

## Supplementary Material

Additional file 1: Figure S1 The HMBC spectrum of the product P1. **Figure S2**. The HMQC spectrum of the product P1. Click here for file

## References

[B1] El-Seedi HR El-Seed AMA Khalifa SAM Görasson U Bohlin L Borg-Karlson A-K Verpoorte R Biosynthesis, natural sources, dietary intake, pharmacokinetic properties, and biological activities of hydroxycinnamic acids J Agri Food Chem 2012 60 44 10877 10895 10.1021/jf301807g 22931195

[B2] Clifford MN Chlorogenic acids and other cinnamates-nature, occurrence and dietary burden J Sci Food Agric 1999 79 3 362 372 10.1002/(SICI)1097-0010(19990301)79:3<362::AID-JSFA256>3.0.CO;2-D

[B3] Herrmann K Occurrence and content of hydroxycinnamic acid and hydoxybenzoic acid compounds in foods Crit Rev Food Sci Nutr 1989 28 4 315 347 10.1080/10408398909527504 2690858

[B4] Crozier A Jaganatha IB Clifford MM Dietary phenolics: chemistry, bioavailability and effects on health Nat Prod Rep 2009 26 8 1001 10043 10.1039/b802662a 19636448

[B5] Chemler JA Koffas MAG Metabolic engineering for plant natural product biosynthesis in microbes Cur Opin Biotech 2008 19 6 597 605 10.1016/j.copbio.2008.10.011 18992815

[B6] Chemler JA Fowler ZL Mchugh KP Koffas MAG Improving NADPH availability for natural product biosynthesis in Escherichia coli by metabolic engineering Metab Eng 2010 12 2 96 104 10.1016/j.ymben.2009.07.003 19628048

[B7] Fowler ZL Koffas MAG Biosynthesis and biotechnological production of flavanones: current state and perspectives Appl Microbiol Biotechnol 2009 83 5 799 808 10.1007/s00253-009-2039-z 19475406

[B8] Beuerle T Pichershy E Enzymatic synthesis and purification of aromatic coenzyme A esters Anal Biochem 2002 302 2 305 312 10.1006/abio.2001.5574 11878812

[B9] Hoffmann L Maury S Martz F Geoffroy P Legrand M Purification, cloning, and properties of an acyltransferase controlling shikimate and quinate ester intermediates in phenylpropanoid metabolism J Biol Chem 2003 278 1 95 103 12381722 10.1074/jbc.M209362200

[B10] Hoffmann L Besseau S Geoffoy P Ritzenthaler C Meyer D Lapierre C Pollet B Legrand M Silencing of hydroxycinnamoyl-Coenzyme A shikimate/quinate hydroxycinnamoyltransferase affects phenylpropanoid biosynthesis Plant Cell 2004 16 6 1446 1465 10.1105/tpc.020297 15161961 PMC490038

[B11] Wagner A Ralph J Akiyama T Flint H Phillips L Torr K Nanayakkara B Kiri LT Exploring lignification in conifers by silencing hydroxycinnamoyl-CoA:shikimate hydroxycinnamoyltransferase in Pinus radiata Proc Natl Aca Sci USA 2007 104 28 11856 11861 10.1073/pnas.0701428104 PMC1913891 17609384

[B12] Comino C Lanteri S Portis E Acquadro A Romani A Hehn A Larbat R Bourgaud F Isolation and functional characterization of a cDNA coding a hydroxycinnamoyltransferase involved in phenylpropanoid biosynthesis in Cynara cardunculus L BMC Plant Biol 2007 20 7 14 17374149 10.1186/1471-2229-7-14 PMC1847684

[B13] Kim B-G Lee ET Ahn J-H Characterization of hydroxycinnamoyl-coenzyme A shikimate hydroxycinnamoyltransferase from Populus euramericana J Kor Soc Appl Biol Chem 2011 54 2 817 821

[B14] St Pierre B De Luca V Romeo JT, Ibrahim R, Varin L, De Luca V Evolution of acyltransferase genes: origin and diversification of the BAHD superfamily of acyltransferases involved in secondary metabolism Recent Advances in Phytochemistry Vol 34. Evolution of Metabolic Pathways 2000 Oxford: Elsevier Science Ltd 285 315

[B15] Horinouchi S Combinatorial biosynthesis of non-bacterial and unnatural flavonoids, stilbenoids and curcuminoids by microorganisms J Antibiot 2008 6 12 709 728 19194030 10.1038/ja.2008.85

[B16] Ikeda M Towards bacterial strains overproducing L-tryptophan and other aromatics by metabolic engineering Appl Microbiol Biotechnol 2006 69 6 615 626 10.1007/s00253-005-0252-y 16374633

[B17] Gosset G Production of aromatic compounds in bacteria Curr Opin Biotech 2009 20 6 651 658 10.1016/j.copbio.2009.09.012 19875279

[B18] Dixon RA Paiva NL Stress-induced phenylpropanoid metabolism Plant Cell 1995 7 7 1085 1097 12242399 10.1105/tpc.7.7.1085 PMC160915

[B19] LeeY J Jeon Y Lee JS Kim B-G Lee CH Ahn J-H Enzymatic synthesis of phenolic CoAs using 4-coumarate:coenzyme A ligase (4CL) from rice Bull Kor Chem Soc 2007 28 3 365 366

[B20] Diaz E Ferrández A Prieto MA Garcia JL Biodegradation of aromatic compounds by Escherichia coli Microbiol Mol Biol Rev 2001 65 4 523 569 10.1128/MMBR.65.4.523-569.2001 11729263 PMC99040

[B21] Jaiswal R Patras MA Eravuchira PJ Kuhnert N Profile and characterization of the chlorogenic acids in green robusta coffee beans by LC-MSn: Identification of seven new classes of compounds J Agric Food Chem 2010 58 15 8722 8737 10.1021/jf1014457 20681662

[B22] Draths KM Knop DR Frost JW Shikimic acid and quinic acid: replacing isolation from plant sources with recombinant microbial biocatalysis J Am Chem Soc 1999 121 7 1603 1604 10.1021/ja9830243

[B23] Escalante A Calderón R Valdivia A de Anda R Hernánde G Ramirez OT Gosset G Bolivar F Metabolic engineering for the production of shikimic acid in an evolved Esherichia coli strain lacking the phosphoenolpyruvate: carbohydrate phosphotransferase system Microbial Cell Fact 2010 9 21 33 10.1186/1475-2859-9-21 PMC2873404 20385022

[B24] Krämer M Bongaerts J Bovenberg R Kremer S Müller U Orf S Wubbolts M Raeven L Metabolic engineering for microbial production of shikimic acid Met Eng 2003 5 4 277 283 10.1016/j.ymben.2003.09.001 14642355

[B25] Niggeweg R Michael AJ Martin C Engineering plants with increased levels of the antioxidant chlorogenic acid Nat Biotech 2004 22 4 746 754 10.1038/nbt966 15107863

[B26] Zhang H Stephanopoulos G Engineering E. coli for caffeic acid biosynthesis from renewable sugar Appl Microbiol Biotechnol 2012 10.1007/s00253-012-4544-8 23179615

[B27] Lindner HA Nadeau G Matte A Michel G Ménard R Cygler M Site-directed mutagenesis of the active site region in the quinate/shimate 5-dehydrogenase YdiB of Escherichia coli J Biol Chem 2005 280 8 7162 7169 10.1074/jbc.M412028200 15596430

[B28] Sung SH Kim BG Ahn J-H Optimization of rhamnetin production in Escherichia coli J Microbio Biotech 2011 21 8 854 857 10.4014/jmb.1104.04048 21876376

[B29] Zheng W Clifford MN Profiling the chlorogenic acids of sweet potato (Ipomoea batatas) from China Food Chem 2010 106 1 147 152

[B30] Garcia-Conesa MT Plumb GW Waldron KW Ralph J Williamson G Ferulic acid dehydrodimers from wheat bran: isolation, purification and antioxidant properties of 8-O-4-diferulic acid Redox Rep 1997 3 5–6 319 323 9754331 10.1080/13510002.1997.11747129

[B31] Berner M Krug D Bihlmaier C Vente A Müller R Bechthold A Genes and enzymes involved in caffeic acid biosynthesis in the actinomycete Saccharothix espanaensis J Bact 2006 188 7 2666 2673 10.1128/JB.188.7.2666-2673.2006 16547054 PMC1428420

[B32] Juminaga D Baidoo EE Redding-Johanson AM Batth TS Burd H Mukhopadhyay A Petzold CJ Keasling JD Modular engineering of L-tyrosine production in Escherichia coli Appl Environ Microbiol 2012 78 1 89 98 10.1128/AEM.06017-11 22020510 PMC3255607

[B33] Michel G Roszak AW Sauvé V Maclean J Matte A Coggins JR Cygler M Lapthorn AJ Structure of shikimate dehydrogenase AroE and its paralog YdiB J Biol Chem 2003 278 21 19463 19472 10.1074/jbc.M300794200 12637497

[B34] Datsenko KA Wanner BL One-step inactivation of chromosomal genes in Escherichia coli K-12 using PCR products Proc Natl Acad Sci USA 2000 97 12 6640 6645 10.1073/pnas.120163297 10829079 PMC18686

[B35] Yoon J-A Kim B-G Lee WJ Lim Y Chong Y Ahn J-H Production of a novel quercetin glycoside through metabolic engineering of Escherichia coli Appl Env Microbiol 2012 78 12 4256 4262 10.1128/AEM.00275-12 22492444 PMC3370505

