# Supplementary material for: Production of hydroxycinnamoyl-shikimates and chlorogenic acid in Escherichia coli: production of hydroxycinnamic acid conjugates
Source: Microb Cell Fact. 2013 Feb 5;12:15. doi: 10.1186/1475-2859-12-15 (PMC3621256; doi:10.1186/1475-2859-12-15)
Supplement: Additional file 1: Figure S1 — The HMBC spectrum of the product P1. Figure S2. The HMQC spectrum of the product P1. [file 1475-2859-12-15-S1.docx]

Supplementary Fig. 1. The HMBC spectrum of the product P1.

Supplementary Figure 2. The HMQC spectrum of the product P1.
